# Metal biomarker mixtures and blood pressure in the United States: cross-sectional findings from the 1999-2006 National Health and Nutrition Examination Survey (NHANES)

**DOI:** 10.1186/s12940-021-00695-1

**Published:** 2021-02-14

**Authors:** Todd M. Everson, Megan M. Niedzwiecki, Daniell Toth, Maria Tellez-Plaza, Haoran Liu, Dana B. Barr, Matthew O. Gribble

**Affiliations:** 1 Gangarosa Department of Environmental Health, Rollins School of Public Health, Emory University, 1518 Clifton Road NE, Mailstop 1518-002-2BB, Atlanta, GA 30322 USA; 2 Department of Environmental Medicine & Public Health, Icahn School of Medicine at Mount Sinai, New York, NY USA; 3 U.S. Bureau of Labor Statistics, Office of Survey Methods Research, D. C, Washington, USA; 4 Biomedical Research Institute INCLIVA, Valencia, Spain; 5 Department of Biostatistics and Bioinformatics, Rollins School of Public Health, Emory University, Atlanta, GA USA; 6 Laboratory for Exposure Assessment and Development for Environmental Research, Department of Environmental Health, Rollins School of Public Health, Emory University, Atlanta, GA 30322 USA; 7 Department of Epidemiology, Rollins School of Public Health, Emory University, 1518 Clifton Road NE, Mailstop 1518-002-2BB, Atlanta, GA 30322 USA

**Keywords:** Mixtures, Risk assessment, Synergy, Antagonism, Survey statistics, cardiovascular epidemiology, Environmental epidemiology

## Abstract

**Background:**

The objective of this study was to identify conditional relationships between multiple metal biomarkers that predict systolic and diastolic blood pressure in the non-institutionalized United States adult population below the age of 60.

**Methods:**

We used inorganic exposure biomarker data and blood pressure data from three cycles (1999–2004) of the National Health and Nutrition Examination Survey (NHANES) to construct regression trees for blood pressure among adults ages 20–60 (adjusted for age, sex, body mass index, race, and smoking status) to identify predictors of systolic (SBP) and diastolic blood pressure (DBP). We also considered relationships among non-Hispanic black, Mexican-American, and white adults separately.

**Results:**

The following metal exposure biomarkers were conditionally predictive of SBP and/or DBP in the full sample: antimony (Sb), barium (Ba), cadmium (Cd), cesium (Cs), lead (Pb), tungsten (W) and molybdenum (Mo). The highest average SBP (> 120 mmHg) was observed among those with low Sb (≤ 0.21 μg/dL) high Cd (> 0.22 μg/g creatinine) and high Pb (> 2.55 μg/dL) biomarkers. Those with the highest average DBP had high urinary W levels (> 0.10 μg/g creatinine) in combination with either urinary Sb > 0.17 μg/g creatinine or those with urinary Sb ≤ 0.17 μg/g creatinine, but with high blood Pb levels (> 1.35 μg/dL). Predictors differed by ethnicity, with Cd as the main predictor of SBP among non-Hispanic black adults, and Pb not selected by the algorithm as a predictor of SBP among non-Hispanic white adults.

**Conclusions:**

Combinations of metal biomarkers have different apparent relationships with blood pressure. Additional research in toxicological experimental models and in epidemiological studies is warranted to evaluate the suggested possible toxicological interactions between Sb, Cd, and Pb; and between W, Sb, and Pb; for cardiovascular (e.g., blood pressure) health. We also think future epidemiological research on inorganic exposure sets in relation to health outcomes like blood pressure might benefit from stratification by race and ethnicity.

**Supplementary Information:**

The online version contains supplementary material available at 10.1186/s12940-021-00695-1.

Elevated blood pressure (BP) contributes greatly to cardiovascular disease morbidity and mortality [1]. In the Global Burden of Disease, Injuries, and Risk Factors study 2015, elevated systolic blood pressure (SBP) was identified as an enormously influential risk factor contributing to disability adjusted life-years worldwide [2]. Elevated BP is positively associated with risk for cardiometabolic diseases, even below the previously established thresholds for hypertension, which has led to lowering the recommended SBP and diastolic BP (DBP) thresholds for defining hypertension by 10 mmHg each [3]. Thus, identifying opportunities to reduce exposure to determinants of higher BP is of substantial public health interest.

The literature examining joint associations between multiple metals and markers of cardiovascular health is limited, particularly in the context of large population-representative samples. There are studies that have considered possible joint effects of metals and metalloids on blood pressure outcomes in the National Health and Nutrition Examination Survey (NHANES), for example zinc-and-copper [4] or mercury-and-selenium [5], but agnostic screens for higher-order interactions between metals for cardiovascular traits in representative population samples are rare. One study [6] used principal components to summarize complex patterns of exposure to multiple metals and relate those principal components to metabolic syndrome in NHANES 2011–2014; however, using principal components emphasizes the variation between people in exposures, irrespective of the health outcomes, and may identify exposure gradients that are different from the set of joint exposures most synergistically relevant for toxicity.

Another study [7] analyzing data from NHANES 2003–2014, applied machine learning approaches that did not directly allow for incorporation of survey design information, in order to generate summary variables (termed “Environmental Risk Scores”) summarizing multiple metals predictive of an oxidative stress biomarker in the study sample; the Environmental Risk Score summary variables were then used as predictors in conventional survey regression analysis with cardiovascular outcomes.

The objective of this study was to investigate whether combinations of metals could predict SBP or DBP in NHANES, which collects rich data on biomarkers of environmental exposures in the US population, using regression trees—a popular data-driven method to identify the conditional effects of multiple exposures. Until recently, regression trees have been inappropriate for analysis of complex population-relevant surveys such as NHANES since traditional regression tree methods do not account for the clustering, stratification and weighting of complex survey designs. This study advances the methodology of population-scale environmental epidemiology by using survey-consistent regression trees to characterize conditional effects of multiple inorganic exposures on blood pressure in NHANES. We recognize that there may be systematic differences in exposure both to metals and to potential effect modifiers by race and ethnicity in the United States, and so in secondary analyses we fitted survey-consistent regression trees within race-ethnicity strata.

## Methods

### Study population

**Fig. 1:**
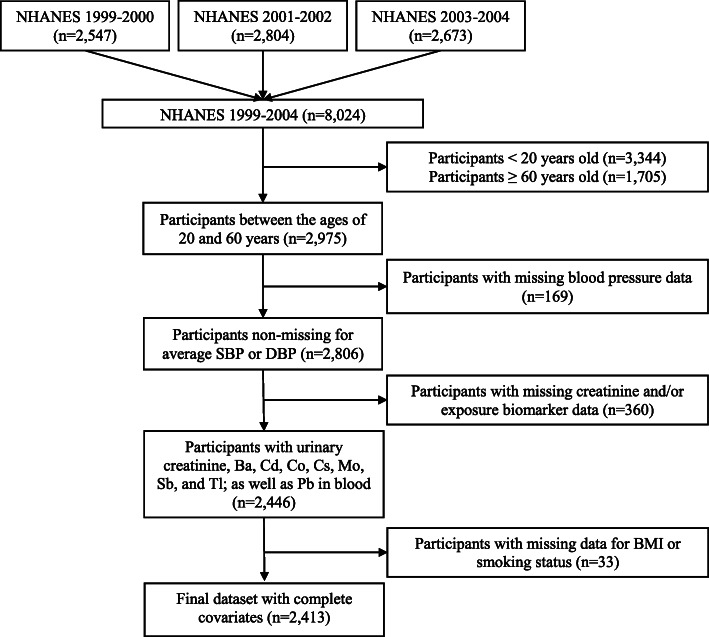
Flow diagram of sample pooling, eligibility, missing data leading to exclusion and final sample used for this analysis

This study was performed using data obtained from NHANES, a nationally-representative cross-sectional survey of the non-institutionalized US population that is conducted by the Centers for Disease Control and Prevention (CDC) on a continuous basis with data-released in two-year cycles. For this analysis, we pooled data from the 1999–2000, 2001–2002, and 2003–2004 survey cycles and restricted our sample to include participants ≥20 years and < 60 years of age who participated at mobile examination centers (MEC), had SBP or DBP measurements taken, and in whom barium (Ba), Cd, Co, Cs, molybdenum (Mo), Sb, thallium (Tl), and tungsten (W) had been measured in urine, and Pb had been measured in blood. We restricted to adults under age 60 because in that decade of age diastolic blood pressure stops increasing and instead begins to decrease [8], and so it is possible the mechanistic biology relevant to blood pressure could be different after that inflection point. Furthermore, since there is some emerging evidence that metabolic syndrome might decrease bone density [9, 10], and lead is accumulated in bone and released into blood as bone density decreases [11, 12], it seemed possible that inclusion of older participants with a higher prevalence of metabolic syndrome [13] in this data analysis could highlight reverse-causal associations driven by possible metabolic syndrome impacts on blood lead concentrations, complicating interpretation of the joint-metal associations. Participants with missing data for body mass index (BMI) or smoking status (*n* = 33) were excluded. One primary sampling unit (SDMVPSU = 3) had very few study participants (*n* = 32) and thus, these participants were pooled with another sampling unit (SDMVPSU = 1) to stabilize model estimation. After pooling data across survey cycles and applying exclusions, our final analytic dataset consisted of 2413 individuals with complete data on metallic concentrations, SBP, DBP, age, sex, race, body mass index (BMI) and smoking status (Fig. 1). We did not correct the study analysis weights for missing data nonresponse so our results might be more comparable with other analyses of NHANES data.

### Systolic and diastolic blood pressure

SBP and DBP were measured via standardized protocols [14, 15]. Briefly, BP was measured in a sitting position after 5 min of rest via a calibrated mercury sphygmomanometer (Baumanometer®) which was fit to the bare right arm. Up to four measures of BP were obtained. If BP was only measured once, that measurement was used, whereas if multiple measures of BP were obtained, the first measure was excluded prior to calculating the average. Averages for systolic blood pressure (SBP) or diastolic blood pressure (DBP) that were recorded in the CDC dataset as zero were recoded as missing.

### Metals concentrations

Blood and urine samples were collected from individuals that participated in the physical exams at MEC; samples were frozen until laboratory analyses could be performed. Urine creatinine was measured using an automatic colorimetric determination based on a modified Jaffe reaction while metals concentrations in urine and blood were measured using inductively coupled plasma-mass spectrometry (ICP-MS) with a dynamic reaction cell to reduce interferences. Urinary cadmium concentrations were adjusted for molybdenum oxide interference [16]. Only the 2003–2004 survey cycle included an indicator variable for urinary and blood metal concentrations below the limits of detection (LOD), so data from this cycle were utilized to identify which metals had high proportions of samples below the LODs. Metals concentrations were detectable in > 80% of samples for all metals, with the exceptions of beryllium (Be) (0.82%), platinum (Pt) (0.47%), Sb (59.44%), and W (78.32%) (Supplemental Table 1); Pt and Be were excluded from this analysis because > 99% of values were below the limit of detection. For all other metals that were below the limit of detection, we utilized the imputed values reported by the CDC, which were equal to the detection limit divided by the square root of two. All metal concentrations measured in urine were standardized for creatinine by dividing each metal by the grams of creatinine per liter of urine. For models in which metals were natural log-transformed, two urinary Cd concentrations that were reported as zero were recoded to the minimum nonzero reported concentrations in our sample (0.00016 μg/g creatinine). Although Pb was measured in both blood and urine, we only included blood Pb concentrations in this analysis since this performs best for both high and low exposure levels [17]. Additionally, Cd was also measured in both blood and urine; we only included urinary Cd in this study since this is more representative of long-term exposure and body burden of Cd [18].

### Confounders and effect modifiers

The potential influences of race and ethnicity, sex, age, body mass index (BMI), and smoking status were controlled for within the survey linear models. We considered that age and BMI might not exhibit linear relationships with SBP or DBP and used loess curves to examine the nature of those associations. The association between both SBP or DBP with age was best approximated by a quadratic function; thus, age and age^2^ were included in the model while the associations with BMI were approximately linear. Race/ethnicity was also considered as a potential effect modifier, and therefore regression trees were also estimated separately within race/ethnicity strata. Tobacco smoke can be a source of exposure for many of these toxic metals [19] and can influence cardiovascular health through other mechanisms [20]. Therefore, tobacco consumption behavior (i.e., current smoker, former smoker, never smoker) was of interest as a confounder of the relationship between joint metal exposures and blood pressure. Participants that reported fewer than 100 cigarettes in their lifetime were coded as ‘never smokers’; those that reported smoking at least 100 cigarettes and that they now smoke cigarettes ‘every day’ or ‘some days’ were coded as ‘current smokers’; and as ‘former smokers’ if they reported that they now smoke ‘not at all’.

### Statistical analyses

To account for the pooling of three different survey cycles, we calculated a 6-year survey weight per the CDC’s recommendation, multiplying the four-year heavy metal subsample weight (WTSHM4YR) by two thirds for participants sampled during 1999–2000 and 2001–2002, while multiplying the two-year subsample weight (WTSA2YR) by one third for participants sampled during 2003–2004. These weights were not further re-calibrated to the subset of complete cases used in our analysis.

Statistical analyses were conducted in R version 3.4.4. We first examined the independent relationship of each individual creatinine-corrected metal, natural log-transformed, with SBP or DBP, while adjusting for age, age^2^, race, sex, BMI, and smoking status, using survey linear regression methods from the survey package [21] and computed Wald-type 95% confidence intervals.

To explore non-linear relationships and potential high-order interactions between metals, we modeled sample-weighted and survey-design corrected regression trees via the recursive partitioning for modelling survey data (*rpms*) method [22, 23]. This method applies a recursive partitioning algorithm to a set of variables to generate a binary regression tree for a continuous outcome. Binary splits are identified using a permutation test that permutes the weighted residuals, *w*_*i*_(*y*_*i*_ − *μ*) within clusters and the estimated cluster effect among clusters, thus accounting for the complex survey design on the expected distribution of Y under the null, where Y is independent of the groups defined by the binary split. For each variable, this test is performed many times, each at the optimal cut-point to account for the fact that the data was observed before selecting splits. Among the variables that are below the designated *p*-value (p-value < 0.05), the variables and the cut-point that leads to the partition with the largest test-statistic is selected to split the data.

Within each node of the tree, the *rpms* algorithm also estimates the parameters of a survey regression model specified by (SBP or DBP ~ age + age^2^ + BMI + sex + race + smoking status), while accounting for the survey design (weights, clusters, and strata), producing survey-design consistent regression models for each node conditional on the binary splits. Predicted means of Y (SBP or DBP in our case) and standard errors are estimated within each node. The recursive partitioning uses the residualized SBP and DBP values within the child nodes until none of the partitioning tests yield permutation *p*-values < 0.05, or when a node reaches a specified size limit. We implemented *rpms* with 2500 permutations and restricted the minimum terminal node size to 10% of our sample size, meaning that the regression tree would not produce end nodes containing fewer than 10% of the unweighted sample. In additional models, we stratified by self-reported non-Hispanic white adults, Mexican American adults, and non-Hispanic black adults to estimate demographically specific regression trees. For each of these demographically specific trees, predicted SBP or DBP were adjusted for age, age^2^, BMI, sex, and smoking status.

## Results

Combining three NHANES survey cycles (1999–2004), we examined how multiple metals were jointly associated with SBP or DBP among the 2413 individuals aged 20–59 years with complete data on SBP, DBP, biomarkers of internal dose of Ba, Cd, Co, Cs, Mo, Pb, Sb, W, and Tl concentrations in urine had been measured (Pb was measured in blood), as well as age, sex, race, BMI, and smoking status. The metals Ba, Cd, Co, Cs, Mo, and Tl were detectable in > 80% of urine samples and Pb was detectable in > 80% of blood samples, per the LODs from the 2003–2004 cycle (Supplemental Table 1); metals that were below the detection limits for > 50% of samples were excluded from this study. The weighted concentrations of each metal overall, and within race/ethnicity specific strata are presented in Table 1.

**Table 1:** Weighted medians and IQRs for metals concentrations in urine (per gram of creatinine) and blood, overall and stratified by race and ethnicity

Median (IQR)	All Samples	Non-Hispanic White Adults	Mexican- American Adults	Non-Hispanic black Adults	Other
Urine (μg/g creatinine)	*N* = 2413	*N* = 1166	*N* = 535	*N* = 509	*N* = 203
Ba	1.33 (0.75, 2.31)	1.45 (0.84, 2.54)	0.99 (0.63, 2.00)	0.90 (0.43, 1.45)	1.33 (0.77, 2.32)
Cd	0.23 (0.13, 0.40)	0.23 (0.13, 0.40)	0.19 (0.12, 0.31)	0.23 (0.13, 0.41)	0.24 (0.16, 0.42)
Co	0.28 (0.19, 0.42)	0.29 (0.20, 0.42)	0.29 (0.20, 0.45)	0.23 (0.16, 0.37)	0.27 (0.20, 0.42)
Cs	4.24 (3.23, 5.67)	4.43 (3.44, 5.90)	4.12 (3.09, 5.22)	3.20 (2.48, 4.08)	4.19 (2.92, 6.00)
Mo	37.3 (25.4, 52.4)	37.6 (25.6, 52.3)	40.6 (29.9, 54.6)	31.7 (21.4, 44.9)	39.5 (25.1, 58.4)
Sb	0.10 (0.06, 0.15)	0.10 (0.06, 0.16)	0.09 (0.07, 0.14)	0.08 (0.06, 0.12)	0.09 (0.06, 0.14)
Tl	0.15 (0.11, 0.20)	0.15 (0.11, 0.21)	0.14 (0.11, 0.18)	0.12 (0.09, 0.17)	0.16 (0.11, 0.21)
W	0.06 (0.03, 0.11)	0.06 (0.04, 0.11)	0.07 (0.04, 0.11)	0.05 (0.03, 0.09)	0.06 (0.03, 0.11)
Blood					
Pb (μg/dL)	1.5 (1, 2.2)	1.4 (1, 2.2)	1.7 (1, 2.7)	1.5 (1.1, 2.4)	1.6 (1, 2.3)

IQR: Inter-quartile range, Ba: barium, Cd: cadmium, Co: cobalt, Cs: cesium, Mo: molybdenum, Pb: lead, Sb: antimony, Tl: thallium, and W: tungsten.

We first examined the correlation structure of the 9 metals. While most metals only exhibited modest, weak, or no correlations, there were a few notable moderate and strong pairwise correlations: cesium (Cs) and thallium (Tl) were strongly correlated (cor = 0.59, *p*-value <2E-16), while barium (Ba) and cobalt (Co), and antimony (Sb) and tungsten (W) were moderately correlated (cor = 0.41 & 0.29, respectively). We then examined the individual associations between metals with SBP or DBP by regressing BP (mm Hg) on the log-transformed metal concentration while adjusting for age, age^2^, sex, race, BMI and smoking status. We observed numerous associations via this individual screening. Higher concentrations of Ba, Sb, W, and Pb were associated with higher SBP, and similarly, though with smaller magnitudes of effect, for DBP; additionally, increasing concentrations of Cs and Mo were associated with lower DBP (Table 2). We then used multiple regression models for both SBP and DBP with all 9 metals included as independent variables and adjusting for the same confounders as in the above models. While adjusting for the concentrations of all other metals, Ba (β_1_ = 1.0, 95% CI = 0.17, 1.84) and Sb (β_1_ = 1.57, 95% CI = 0.19, 2.95) were associated with elevated SBP, while Mo (β_1_ = − 1.42, 95% CI = − 2.69, − 0.15) was associated lower SBP. Additionally, while adjusting for all other metals, W (β_1_ = 0.65, 95% CI = 0.03, 1.27) was associated with elevated DBP and Mo (β_1_ = − 1.44, 95% CI = − 2.10, − 0.77) was associated with lower DBP.

**Table 2:** Estimated change in SBP (mm Hg) and DBP (mm Hg) corresponding to a log increase in metals concentrations, adjusted for age, age^2^, race, sex, BMI, and smoking status; parameters estimated via survey linear regression. Models were fit separately for each metal

	SBP (mm Hg)				DBP (mm Hg)			
Metals	β_**1**_	SE	P-value	95% CI	β_**1**_	SE	P-value	95% CI
Urine								
Ba	1.20	0.41	0.0065	(0.38, 2.00)	0.47	0.31	0.1428	(−0.1, 1.07)
Cd	−0.23	0.56	0.6876	(−1.3, 0.86)	0.14	0.32	0.6706	(− 0.4, 0.76)
Co	0.99	0.58	0.0977	(−0.1, 2.13)	0.08	0.33	0.8042	(−0.5, 0.73)
Cs	−1.13	0.88	0.2083	(−2.8, 0.59)	−1.49	0.67	0.0321	(−2.7, −0.1)
Mo	−1.06	0.60	0.0876	(− 2.2, 0.12)	−1.34	0.32	0.0002	(−1.9, −0.7)
Sb	2.09	0.60	0.0013	(0.92, 3.25)	1.04	0.49	0.0421	(0.07, 2.01)
Tl	−0.26	0.98	0.7939	(−2.1, 1.66)	−1.18	0.59	0.0516	(−2.3, −0.0)
W	1.05	0.43	0.0207	(0.20, 1.89)	0.62	0.31	0.0568	(0.00, 1.23)
Blood								
Pb	1.39	0.68	0.0478	(0.06, 2.71)	0.78	0.49	0.1197	(−0.1, 1.73)

SBP: systolic blood pressure, DBP: diastolic blood pressure, BMI: body mass index, β_1_: slope of the regression line, SE: standard error, CI: confidence interval, Ba: barium, Cd: cadmium, Co: cobalt, Cs: cesium, Mo: molybdenum, Pb: lead, Sb: antimony, Tl: thallium, and W: tungsten.

**Fig. 2:**
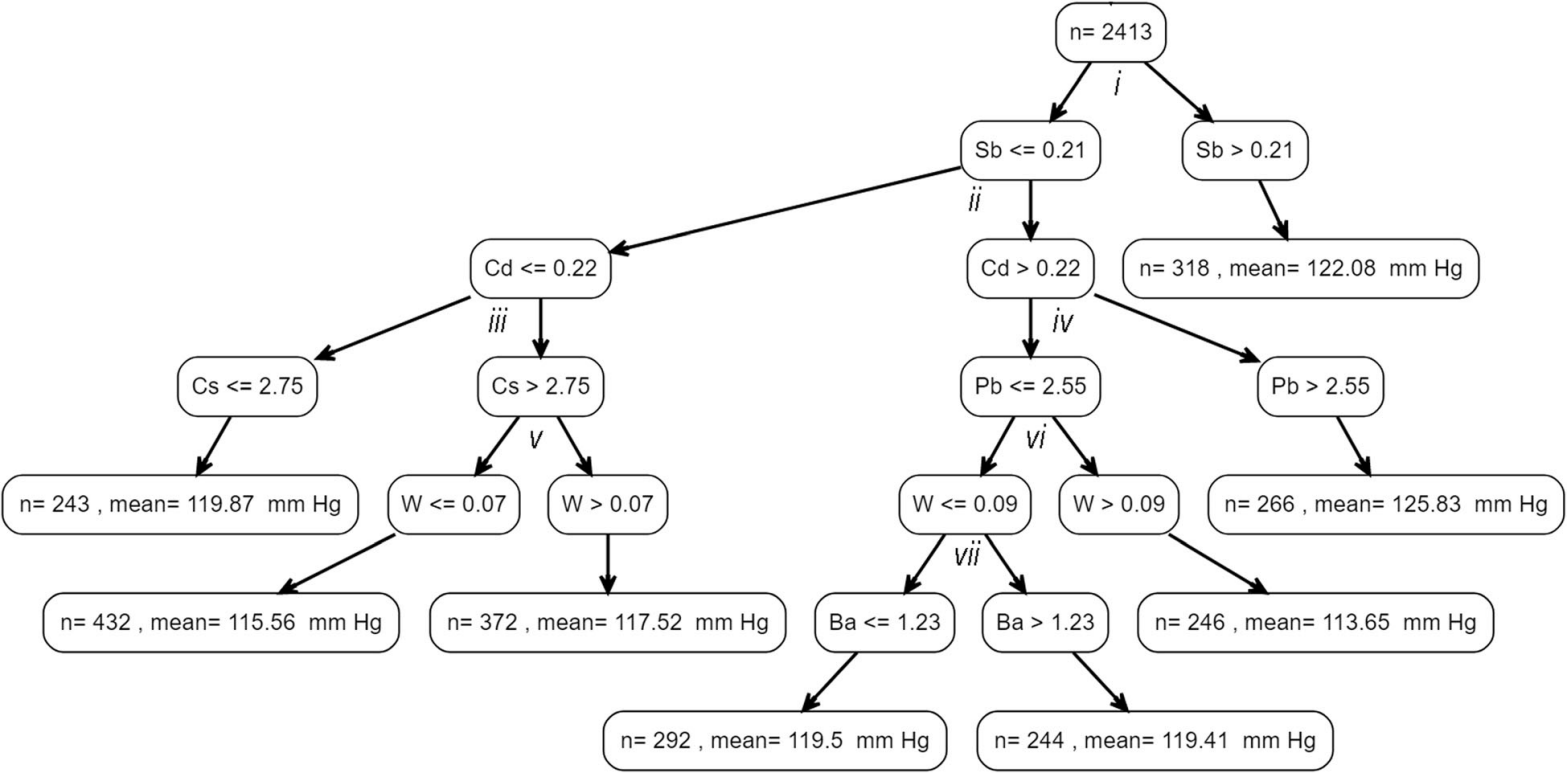
Regression tree for estimated average SBP as a product of metals biomarkers in urine and blood while adjusting for age, age^2^, sex, race, BMI, and smoking status; partitions are labeled (i-vii) so that the difference in SBP that is acheived with each binary split can be observed in Table 3. Concentrations of metal biomarkers are either μg/dL blood (Pb only) or μg/g creatinine (all other metals in urine)

**Fig. 3:**
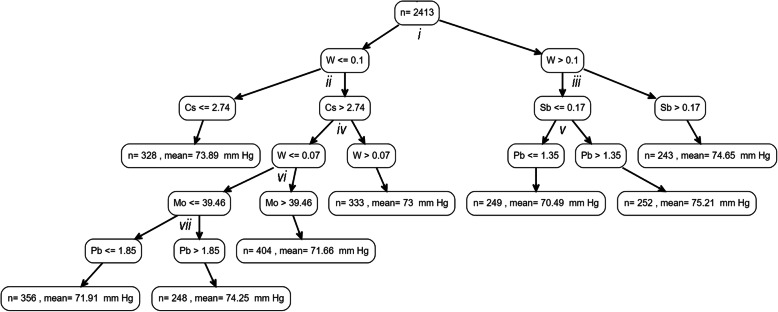
Regression tree for estimated average DBP as a product of metals biomarkers in urine and blood while adjusting for age, age^2^, sex, race, BMI, and smoking status; partitions are labeled (i-vii) so that the difference in DBP that is acheived with each binary split can be observed in Table 4. Urine metal biomarkers are corrected for grams of creatinine. Concentrations of metal biomarkers are either μg/dL blood (Pb only) or μg/g creatinine (all other metals in urine)

We then applied the *rpms* algorithm to examine conditional relationships of metals biomarker combinations with SBP or DBP, while adjusting for age, age^2^, race, sex, BMI, and smoking status. This method identified Ba, Cd, Cs, Pb, Sb, and W as potential predictors of SBP (Fig. 2 & Table 3) and Cs, Mo, Pb, Sb, and W as the predictors of DBP (Fig. 3 & Table 4). Sb was selected in the root node for the SBP tree, and those with high urinary Sb (> 0.21 μg/g creatinine) had the second highest SBP overall (122.08 mmHg). Those with the highest estimated SBP (125.83 mmHg) had Sb ≤ 0.21 μg/g creatinine, but with high urinary Cd (> 0.22 μg/g creatinine) and high blood Pb (> 2.55 μg/dL).

**Table 3:** Estimated mean SBP (mm Hg) within each node produced from binary partitions from metals concentrations, adjusted for age, age^2^, race, sex, BMI, and smoking status; partitions can be linked to the SBP tree via lower case roman numerals (*i*-*vii*), and child nodes are classified as left (L) or right (R), relative to their position in the tree, for each partition

Partition	Node	End Node	Splits	N	Mean SBP
NA	Root	No	NA	2413	119.05
*i*	L	No	Sb ≤ 0.21	2095	118.59
	R	Yes	Sb > 0.21	318	122.08
*ii*	L	No	Cd ≤ 0.22	1047	117.34
	R	No	Cd > 0.22	1048	119.74
*iii*	L	Yes	Cs ≤ 2.75	243	119.87
	R	No	Cs > 2.75	804	116.53
*iv*	L	No	Pb ≤ 2.55	782	117.82
	R	Yes	Pb > 2.55	266	125.83
*v*	L	Yes	W ≤ 0.07	432	115.56
	R	Yes	W > 0.07	372	117.52
*vi*	L	No	W ≤ 0.09	536	119.59
	R	Yes	W > 0.09	246	113.65
*vii*	L	Yes	Ba ≤1.23	292	119.50
	R	Yes	Ba > 1.23	244	119.41

SBP: systolic blood pressure, Ba: urinary barium (μg/g creatinine), Cd: urinary cadmium (μg/g creatinine), Cs: urinary cesium (μg/g creatinine), Pb: blood lead (μg/dL), Sb: urinary antimony (μg/g creatinine), W: urinary tungsten (μg/g creatinine).

**Table 4:** Estimated mean DBP (mm Hg) within each node produced from binary partitions from metals concentrations, adjusted for age, age^2^, race, sex, BMI, and smoking status; partitions can be linked to the DBP tree via lower case roman numerals (*i*-*vii*), and child nodes are classified as left (L) or right (R) for each partition

Partition	Node	End Node	Splits	N	Mean DBP
NA	Root	No	NA	2413	73.06
*i*	L	No	W ≤ 0.1	1669	72.85
	R	No	W > 0.1	744	73.48
*ii*	L	Yes	Cs ≤ 2.74	328	73.89
	R	No	Cs > 2.74	1341	72.59
*iii*	L	No	Sb ≤ 0.17	501	72.83
	R	Yes	Sb > 0.17	243	74.65
*iv*	L	No	W ≤ 0.07	1008	72.43
	R	Yes	W > 0.07	333	73.00
*v*	L	Yes	Pb ≤ 1.35	249	70.49
	R	Yes	Pb > 1.35	252	75.21
*vi*	L	No	Mo ≤ 39.46	604	72.96
	R	Yes	Mo > 39.46	404	71.66
*vii*	L	Yes	Pb ≤ 1.85	356	71.91
	R	Yes	Pb > 1.85	248	74.25

DBP: diastolic blood pressure, Cs: urinary cesium (μg/g creatinine), Mo: urinary molybdenum (μg/g creatinine), Pb: blood lead (μg/dL), Sb: urinary antimony (μg/g creatinine), W: urinary tungsten (μg/g creatinine).

We also fitted trees separately for non-Hispanic black adults, Mexican American adults, and non-Hispanic white adults. Among non-Hispanic black adults (Supplemental Fig. 1), Cd was selected as the root node (> 0.36 μg/g creatinine) for SBP. Among non-Hispanic black adults, higher Pb levels were associated with higher SBP, conditional on concentrations of Cd and Mo. Among Mexican American adults (Supplemental Fig. 2), Cs was selected as the root node for SBP, and the combination of high Cs (> 3.90 μg/g creatinine) with high blood Pb (2.45 μg/dL) was associated with the highest average SBP (125.26 mmHg). Among non-Hispanic white adults (Supplemental Fig. 3), the combination of high W (> 0.10 μg/g creatinine) with high Sb (> 0.21 μg/g creatinine) was associated with the highest average SBP (122.05 mmHg). Interestingly, blood Pb was not selected as a risk factor for elevated SBP among non-Hispanic white adults, however it was a predictor of SBP among Mexican American adults (Supplemental Fig. 2) and non-Hispanic black adults (Supplemental Fig. 1).

The patterns were slightly different for DBP. Among non-Hispanic black adults (Supplemental Fig. 4), higher Pb (> 1.65 μg/dL) was a predictor of high DBP only among those with Cs ≤ 2.36 μg/g creatinine. The DBP tree for Mexican American adults (Supplemental Fig. 5) identified Cs and W as predictors, in which lower levels of these metals were associated with lower DBP, conditional on Tl concentrations. Among non-Hispanic white adults (Supplemental Fig. 6) Pb was selected as the root node.

Finally, we tested whether the regression trees for SBP and DBP that were trained on three NHANES cycles (1999–2000, 2001–2002, and 2003–2004), could be informative about SBP and/or DBP in an independent NHANES dataset (the 2005–2006 cycle). For this analyses, we predicted SBP and DBP for each participant in the 2005–2006 cycle with complete biomarker, blood pressure, and covariate data while applying the same exclusion criteria (*n* = 950) using the primary regression trees that were trained on the 1999–2004 cycles, then compared predicted versus reported blood pressure with Spearman’s correlations. We found that that predicted SBP and DBP were moderately positively correlated with reported SBP and DBP (rho_SBP_ = 0.41 and rho_DBP_ = 0.37, *p*-values < 2.2E-16) in these independent data.

## Discussion

Our study found that increased biomarker concentrations of Ba, Sb, W and Pb exposure were associated with elevated SBP, Sb was also associated with elevated DBP, and biomarkers of Cs and Mo were inversely associated with DBP in linear regression models fit for each metal separately. While controlling for all metal concentrations simultaneously, four metals (Ba, Sb, W, and Mo) were associated with SBP and DBP. When we applied the regression tree approach that accommodates complex joint effects of multiple metal exposures, many of the metals predictive of SBP or DBP in this analysis had substantial overlap with the set identified in the more traditional regression approach. Those with higher concentrations of Sb, Cd, W, and Pb tended to have higher predicted SBP, while those with higher Cs and Mo tended to have lower SBP and DBP.

The metal that was most strongly associated with SBP (Sb) in linear models was also selected by the *rpms* algorithm as the metal on which to partition the root node for the SBP tree, suggesting that Sb is an important overall predictor of SBP at the population level. However, the regression tree approach provided additional insights regarding the joint relationships between metals and blood pressure. For instance, at the population-level, it appears as though Sb, Cd, Pb, and their potential interactions may be particularly important for elevated systolic blood pressure. We found that the only sub-groups with average SBP estimated to be greater than 120 mmHg, were those with either Sb > 0.21 μg/g creatinine, or those with Sb ≤ 0.21 μg/g creatinine but in combination with Cd > 0.22 μg/g creatinine and with blood Pb > 2.55 μg/dL. Additionally, the highest estimated DBP was among those with a metal biomarker profile of W > 0.10 μg/g creatinine, Sb ≤ 0.17 μg/g creatinine, and blood Pb > 1.35 μg/dL. Although, Cd and Pb have previously been characterized as risk factors for higher blood pressure, there is less evidence regarding the potential impacts of W and Sb [24], and none explicitly examining their interactions. Our findings suggest that further research is needed to better characterize the impacts of W and Sb on cardiovascular health, and their potential interactions with Cd and Pb concentrations.

Our analysis also suggested that the metallic determinants of SBP and DBP differ across demographic subgroups of the US population, though there are also some similarities. For instance, blood Pb levels were identified as a predictor of elevated SBP in both non-Hispanic black adults and Mexican American adults, and of elevated DBP among both non-Hispanic white adults and non-Hispanic black adults. However, the conditional relationships between Pb and other metals for their impacts on BP differed within each of these sub-groups. Similarly, W was included in all SBP and DBP trees, except for the DBP tree for non-Hispanic black adults, and was often selected included within the same branch of the tree as Pb. Antimony (Sb), which was the root node for the population-level SBP tree, appears to primarily affect SBP among non-Hispanic white adults, while Cd appears to be a particularly important predictor of elevated SBP among non-Hispanic black adults. The unique metallic risk factors and conditional relationships within these racial and ethnic subgroups could be due to different sources of exposure. For example: a major source of cadmium exposure is smoking [25], and non-Hispanic black Americans are more likely to smoke menthol-flavored cigarettes than white or Mexican-American adults [26], so the uniquely significant relationships of blood pressure to cadmium in non-Hispanic black adults might be reflecting exposures related to menthol cigarettes and menthol cigarette smoking-related behaviors. A limitation of our study is that we only considered the differences in conditional inference trees by race/ethnicity and did not account for possible differences in tree structure according to socioeconomic position independent of race and ethnicity. Both race/ethnicity and socioeconomic position play important roles in shaping exposure to environmental hazards in the United States [27–29].

Our study provides significant and novel insights into how environmental exposure to metals relate to blood pressure in the United States population. The most notable results from our study were the identification of thresholds and joint relationships between W, Sb, Cd, and Pb as determinants of elevated SBP and DBP. Exposure to Pb and Cd in the US population has declined since the 1980s, and a recent study found that the declining rates of cardiovascular diseases are likely related, in part, to these reductions in exposure to Pb and Cd [30]. However, since Pb and Cd have been often related to cardiovascular outcomes and elevated blood pressure [31–33], if they are toxic even at low doses [34], then further reductions in exposure to these two metals might benefit public health.

Our results suggest that higher Sb concentrations were associated with higher SBP, and that co-exposure to Cd and Pb, among those with lower Sb concentration, may have elevated SBP. We also found that higher W concentrations were associated with higher DBP, and our observed associations with Sb and Pb on DBP were conditional on W concentrations. Although numerous epidemiologic studies have observed relationships between Cd and Pb with elevated blood pressure [24], fewer studies have examined the potential cardiovascular impacts of Sb or W. However, elevated Sb exposure has been associated with increased prevalence of heart disease and heart disease mortality in the US population between 1999 and 2010 [35] and associated with high BP, defined as ≥140 or 90 mmHg for SBP or DBP, respectively, in the 2011–2012 NHANES survey cycle [36]. W and Sb were also associated in NHANES 1999–2006 with a composite outcome of cardiovascular and cerebrovascular disease [37]. Our findings complement these studies, indicating that Sb and W may be predictors of elevated BP. Unlike these past studies, we found that W and Sb may also modify the relationships between Pb and Cd with BP.

Our findings also suggest that higher concentrations of Cs and Mo were associated with lower BP (Cs: lower SBP, Cs and Mo: lower DBP). Previous studies examining the impacts of Cs and Mo on BP have been limited in number. Our current results support findings from a previous analysis of NHANES survey cycle data from 1999 to 2012, which found that Cs was associated with lower SBP and DBP [38], and results from a small epidemiological study of particulate matter (PM) constituents, which found negative associations between Mo and SBP/DBP [39]. Two studies in rat models also support that exposure to Cs [40] and Mo [41] may reduce BP. However, results from other studies have yielded different conclusions: two NHANES analyses (2009–2012 and 2011–2012 cycles) identified positive associations of Cs and Mo with high BP [36, 42], and a study of aging men with metabolic syndrome found no significant Spearman correlation between urinary Mo and SBP or DBP [43]. Additional studies are warranted to examine the relationships of Sb and Mo with BP.

This paper illustrates a novel data analysis approach that can be applied to other studies of how multiple exposures may associate with health outcomes, while leveraging the rich exposure data in large surveys. Though our analysis focused on a panel metals, this approach can complement the growing interest in environment-wide and exposure-mixture studies [44] to include exploration of how joint-associations between multiple co-exposures and components of mixtures can impact health. However, there are some limitations to this approach that cannot be overlooked. First, as with any modeling effort, the quality of results is dependent on the strength of the input data. Regression trees are entirely data-driven approaches that have the potential to overfit, meaning that the prediction tree may explain more of the variance in the supplied data than it would in an independent dataset. There are numerous tree-based ensemble methods designed to reduce overfitting by resampling both predictors and observations to produce multiple regression or classification trees, most notably random forests [45] and gradient-boosted trees [46]. These methods produce more accurate predictions, but interpretation of the actual relationships between predictors is not possible. Ensemble methods for regression trees have not yet been developed that are suitable for complex survey data analysis, and this is an area of interest for future methods development.

## Conclusions

In summary, in this application of *rpms* to data from adults age 20–60 in the United States, we identified numerous relationships between metal exposure biomarkers and SBP or DBP, with the most notable combinations being the potential interactions between W, Sb, Cd and Pb. These findings add to the current body of evidence that Cd and Pb exposures are risk factors for elevated blood pressure, while providing additional evidence for an association between Sb and W with elevated BP, which may also modify the cardiovascular impacts of Pb and Cd. We also found that racial and ethnic sub-groups of the US population may have different metallic determinants of SBP and DBP, with high Sb being particularly relevant among non-Hispanic white adults and Cd being especially pertinent for BP among non-Hispanic black adults. The *rpms* framework can be used to model additional sets of exposures in future studies, advancing understanding of how the environment in gestalt contributes to health and disease in specific (formally surveyed) human populations.

## Supplementary Information

**Additional file 1: Supplemental Fig. 1.** Among non-Hispanic black adults: regression tree for estimated SBP as a product of metals biomarkers in urine and blood while adjusting age, age^2^, sex, BMI and smoking status. Urine metal biomarkers are corrected for grams of creatinine. Concentrations of metal biomarkers are either μg/dL blood (Pb only) or μg/g creatinine (all other metals in urine).

**Additional file 2: Supplemental Fig. 2.** Among Mexican-American adults: regression tree for estimated SBP as a product of metals biomarkers in urine and blood while adjusting age, age^2^, sex, BMI and smoking status. Urine metal biomarkers are corrected for grams of creatinine. Concentrations of metal biomarkers are either μg/dL blood (Pb only) or μg/g creatinine (all other metals in urine).

**Additional file 3: Supplemental Fig. 3** Among non-Hispanic white adults: regression trees for estimated SBP as a function of metals biomarkers in urine and blood while adjusting age, age^2^, sex, BMI and smoking status. Concentrations of metal biomarkers are either μg/dL blood (Pb only) or μg/g creatinine (all other metals in urine).

**Additional file 4: Supplemental Fig. 4.** Among non-Hispanic black adults: regression tree for estimated DBP as a function of metals biomarkers in urine and blood while adjusting age, age^2^, sex, BMI and smoking status. Concentrations of metal biomarkers are either μg/dL blood (Pb only) or μg/g creatinine (all other metals in urine).

**Additional file 5: Supplemental Fig. 5.** Among Mexican-American adults: regression tree for estimated DBP as a function of metals biomarkers in urine and blood while adjusting age, age^2^, sex, BMI and smoking status within strata of Mexican American adults. Concentrations of metal biomarkers are either μg/dL blood (Pb only) or μg/g creatinine (all other metals in urine).

**Additional file 6: Supplemental Fig. 6.** Among non-Hispanic white adults: regression tree for estimated DBP as a function of metals biomarkers in urine and blood while adjusting age, age^2^, sex, BMI and smoking status. Concentrations of metal biomarkers are either μg/dL blood (Pb only) or μg/g creatinine (all other metals in urine).

**Additional file 7: Supplemental Table 1.** Proportions of metals concentrations with reported values that were above the limits of detection from the 2003-2004 cycle (n=852).

**Additional file 8:** Analysis Scripts and Data.

## Data Availability

This work uses publicly available datasets, and our code for accessing and analyzing these data is provided in the Supplement.
