# Supplementary material for: Metal biomarker mixtures and blood pressure in the United States: cross-sectional findings from the 1999-2006 National Health and Nutrition Examination Survey (NHANES)
Source: Environ Health. 2021 Feb 14;20:15. doi: 10.1186/s12940-021-00695-1 (PMC7883578; doi:10.1186/s12940-021-00695-1)
Supplement: Supplementary file 7 — Additional file 7: Supplemental Table 1. Proportions of metals concentrations with reported values that were above the limits of detection from the 2003-2004 cycle (n=852). [file 12940_2021_695_MOESM7_ESM.docx]

**Supplemental Table 1:** Proportions of metals concentrations with reported values that were above the limits of detection from the 2003-2004 cycle (n=852).

| Metal | Detectable (%) |
| --- | --- |
| Urine |  |
| Ba | 790 (93%) |
| Be | 7 (< 1%) |
| Cd | 801 (94%) |
| Co | 804 (94%) |
| Cs | 852 (100%) |
| Mo | 849 (>99%) |
| Pt | 3 (< 1%) |
| Sb | 504 (59%) |
| Tl | 844 (99%) |
| W | 667 (78%) |
| Blood |  |
| Pb | 849 (>99%) |

Ba: barium, Be: beryllium, Cd: cadmium, Co: cobalt, Cs: cesium, Mo: molybdenum, Pb: lead, Pt: platinum, Sb: antimony, Tl: thallium, and W: tungsten.
