# Supplementary material for: Metal biomarker mixtures and blood pressure in the United States: cross-sectional findings from the 1999-2006 National Health and Nutrition Examination Survey (NHANES)
Source: Environ Health. 2021 Feb 14;20:15. doi: 10.1186/s12940-021-00695-1 (PMC7883578; doi:10.1186/s12940-021-00695-1)

Supplemental Figure 3: Among non-Hispanic white adults: regression trees for estimated SBP as a function of metals biomarkers in urine and blood while adjusting age, age^2, sex, BMI and smoking status. Concentrations of metal biomarkers are either µg/dL blood (Pb only) or µg/g creatinine (all other metals in urine).

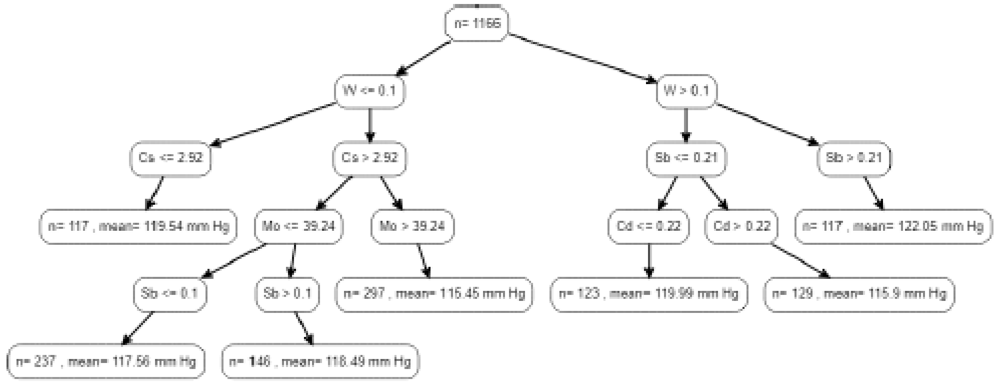

Supplement: Supplementary file 4 [file 12940_2021_695_MOESM3_ESM.docx]
